# Serial Severity Assessment in Advanced Stages of AMI-Cardiogenic Shock Requiring Micro-axial Flow Pump Support

**DOI:** 10.1016/j.jscai.2025.102565

**Published:** 2025-03-28

**Authors:** Chirag Mehta, Phinnara Has, Brian Osorio, Mark Godding, Vishal Khetpal, Annaliese Clancy, Aryan Mehta, Athena Poppas, Rachna Kataria, Marwan Saad, Omar N. Hyder, Adeel A. Abbasi, Neel R. Sodha, Mark J. Cunningham, J. Dawn Abbott, Saraschandra Vallabhajosyula

**Affiliations:** a Department of Medicine, The Warren Alpert Medical School of Brown University, Providence, Rhode Island; b Biostatistics, Epidemiology, Research Design, and Informatics Team, Brown University Health, Providence, Rhode Island; c Division of Cardiology, Department of Medicine, The Warren Alpert Medical School of Brown University, Providence, Rhode Island; d Department of Pharmacy, Brown University Health Rhode Island Hospital, Providence, Rhode Island; e Brown University Health Cardiovascular Institute, Providence, Rhode Island; f Department of Medicine, University of Connecticut School of Medicine, Farmington, Connecticut; g Division of Pulmonary, Critical Care, and Sleep Medicine, Department of Medicine, The Warren Alpert Medical School of Brown University, Providence, Rhode Island; h Division of Cardiothoracic Surgery, Department of Surgery, The Warren Alpert Medical School of Brown University, Providence, Rhode Island

**Keywords:** acute myocardial infarction, cardiogenic shock, mechanical circulatory support, serial assessment, vasoactive medications

Cardiogenic shock complicating acute myocardial infarction (AMI-CS) is a hemodynamically complex, heterogeneous syndrome associated with high morbidity and mortality (30%-50%) despite advances in acute cardiovascular care.^1^ Coronary revascularization and the use of a micro-axial flow pump (mAFP) (Impella CP, Abiomed) have been associated with improved outcomes in AMI-CS clinical trials^1^; however, only 32% of ST-segment-elevation AMI-CS patients in practice meet DanGer Shock (Danish-German Cardiogenic Shock) trial enrollment criteria, which include early presentation, absence of comatose cardiac arrest, and only left ventricular involvement.^2^ In addition, analyses of large national data sets found that only 1.5% of patients who receive a mechanical circulatory support (MCS) device get subsequent escalation, and in those who do, median time to escalation is nearly 2 days.^3^ In patients with AMI-CS, insertion of MCS may alleviate the deleterious impact of vasoactive medications.^4^ There are, however, limited data on serial vasoactive-inotropic scores (VIS) and hemodynamics following device insertion in patients with advanced cardiogenic shock (Society for Cardiovascular Angiography & Interventions [SCAI] stages D and E).

In this study, we sought to assess the impact of mAFP devices on hemodynamics and vasoactive medication burden in SCAI stage D/E AMI-CS. All adult (≥18 years) AMI-CS patients presenting with SCAI D and E cases treated with Impella CP support from January 1, 2017, to December 31, 2023, at our tertiary cardiovascular care system were enrolled. Brown University Health is a 5-hospital system, consisting of 2 hospitals with cardiac catheterization laboratories and 24 × 7 primary percutaneous coronary intervention (PCI) capability. Both hospitals can perform the full-spectrum of PCI therapies and mAFP placement, and 1 hospital has cardiac surgical, veno-arterial extracorporeal membrane oxygenation (VA-ECMO), and surgical MCS (Impella 5.5, CentriMag) capabilities. This hospital system does not have the ability to implant durable MCS or cardiac transplantation but has a well-established pathway to a regional center for such therapies. Baseline variables, hemodynamics, PCI details, cardiac intensive care unit interventions, and complication rates were collected using electronic medical record data sets. Using previously validated methods, VIS and invasive hemodynamics were calculated every 4 hours for the first 24 hours, unless there was a subsequent device upgrade or patient death.^4^ Patients receiving mAFP support for left ventricular venting in VA-ECMO and heart failure CS were excluded. Data are presented as medians (IQR) and were compared using repeated Friedman tests. A 2-tailed *P* ≤ .05 was considered statistically significant. Institutional review board approval for retrospective study without the need for informed consent was obtained (Brown University Health Institutional Review Board #2084273-3), and appropriate ethical guidelines were followed.

**Figure 1:**
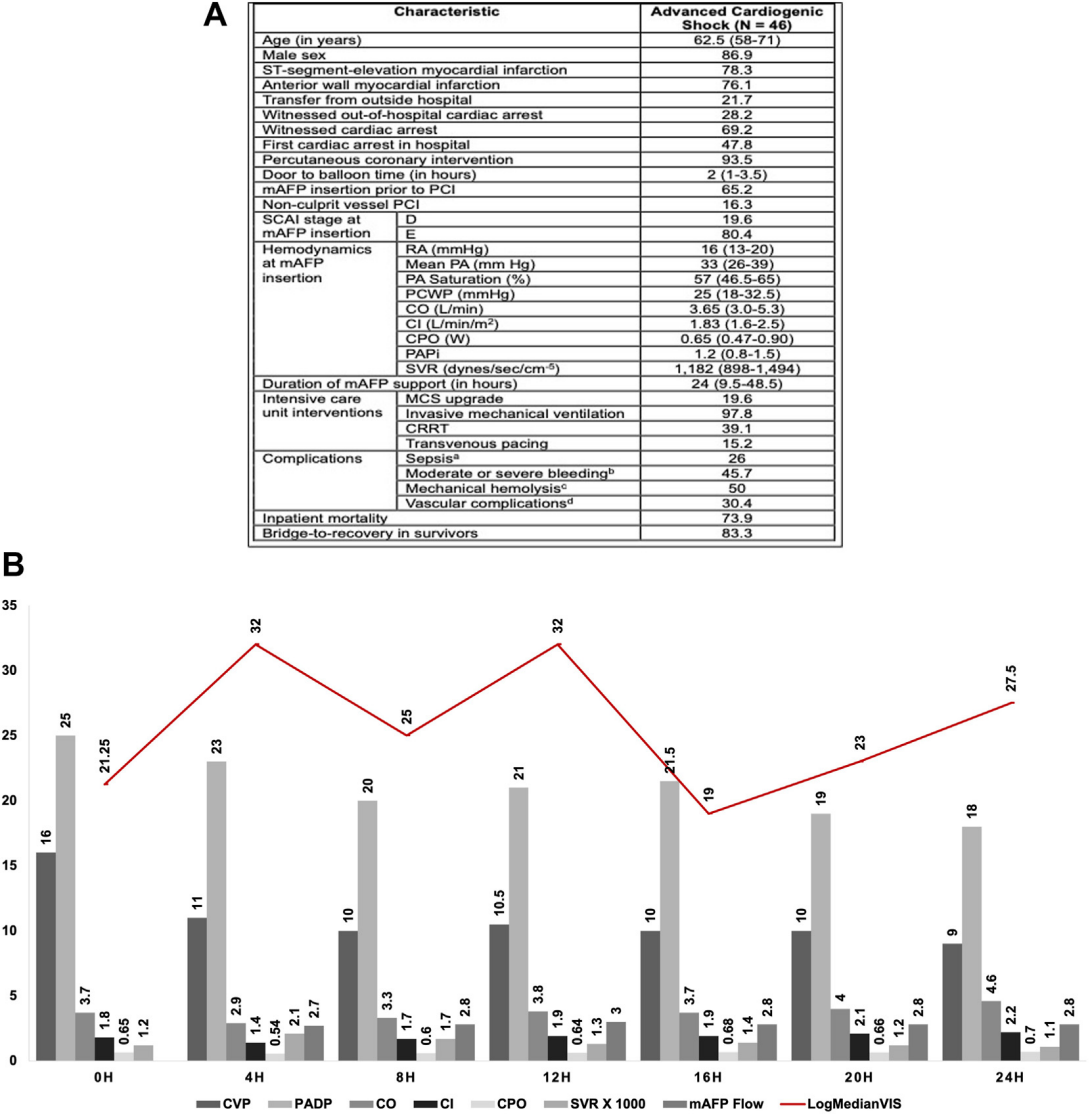
**Study population.** (A) Baseline, hemodynamic, angiographic, and intensive care unit characteristics of advanced AMI and CS patients. (B) Temporal trends of invasive hemodynamics and LogMedianVIS during the first 24 hours in SCAI stage D/E AMI-CS. Data represented as percentage or median (IQR). ^a^Defined as meeting standard systemic inflammatory criteria with evidence of bacteremia or fungemia. ^b^Per International Society for Thrombosis and Hemostasis guidelines. ^c^Defined as plasma free hemoglobin concentration >20 mg/dL or a serum lactate dehydrogenase level >2.5 times the upper normal range. ^d^Acute limb ischemia, compartment syndrome, femoral aneurysm or pseudoaneurysm, and requirement for external femoral-femoral bypass. AMI, acute myocardial infarction; CI, cardiac index; CO, cardiac output; CPO, cardiac power output; CRRT, continuous renal replacement therapy; CS, cardiogenic shock; CVP, central venous pressure; mAFP, micro-axial flow pump; MCS, mechanical circulatory support; PA, pulmonary artery; PADP, pulmonary artery diastolic pressure; PAPi, pulmonary artery pulsatility index; PCI, percutaneous coronary intervention; PCWP, pulmonary capillary wedge pressure; RA, right atrium; SCAI, Society for Cardiovascular Angiography and Intervention; SVR, systemic vascular resistance; VIS, vasoactive inotrope score.

In this 7-year study, 46 patients with AMI-CS in SCAI stage D/E at the time of mAFP insertion were identified (median age 62.5 years; 86.9% males). ST-segment-elevation AMI and anterior wall involvement were noted in 78.1% and 76.1%, respectively. PCI was performed in 93.5%, of which 65.1% received left ventricular unloading prior to intervention (Figure 1A). At the time of mAFP insertion, median cardiac index was 1.83 (IQR, 1.6-2.5) L/min/m^2^ (32.6% pre-mAFP, 54.3% post-mAFP, 6% not measured) with elevated biventricular filling pressures, and 80.4% were in SCAI stage E, with a median VIS of 21.25 (IQR, 10-44). Median duration of mAFP support was 24 (IQR, 9.5-48.5) hours. During the subsequent 24 hours, invasive hemodynamic data and VIS were noted for whom these data were available. The biventricular filling pressures, cardiac output, and cardiac power output largely remained unchanged despite the implantation of an mAFP without any differences between those with and without concomitant cardiac arrest (Figure 1B). Further, use of mAFP did not decrease vasoactive medication burden, measured as LogMedianVIS (Figure 1B). Approximately 20% of our population required escalation to VA-ECMO, and complications were noted in approximately 50% of the patients. In-hospital mortality in this population was 73.9%, with no differences based on presence of cardiac arrest (81.8% vs 62.5%; *P* = .20). Of the survivors, 83.3% were bridged from MCS to recovery, and the remainder were transferred to the partnered quaternary care center for durable MCS or cardiac transplantation evaluation.

In this single-center retrospective study, we found that the use of mAFP in SCAI D and E AMI-CS was not associated with improvements in hemodynamic status or vasoactive medication requirements. In-hospital mortality and the burden of end-organ failure remained high.

Our findings can be explained by multiple hypotheses. First, patients with SCAI stage D/E CS have severe deterioration of cardiac output/index due to pump failure and may often be in advanced stages of cardiometabolic failure with end-organ injury and failure.^5^ Given the severity of ventricular dysfunction, these patients can have loss of pulsatility on mAFP devices, which has been associated with worse outcomes in AMI-CS patients. Second, despite the implantation of mAFP, these patients had continued need for high-dose pharmacological support, which by itself has been associated with worse in-hospital outcomes independent of SCAI stage and MCS utilization.^4^ Furthermore, the use of mAFP in association with high doses of vasoactive medications may result in significantly higher vascular and limb complications, which are associated with greater short-term mortality. Third, it is possible that in those with overlap of cardiac arrest and CS (nearly 50% of our population), neurological injury rather than circulatory failure was driving the clinical outcomes and potentially treatment-limiting decisions. However, we did not note any significant differences in outcomes in SCAI stage D/E AMI-CS patients with and without concomitant cardiac arrest. Lastly, mAFP insertion was at the discretion of the treating operator without specific protocols for device insertion, escalation, or timing, which may confound our findings.

In patients with advanced stages of AMI-CS, a percutaneously implanted mAFP as the initial MCS strategy was insufficient to improve either hemodynamic indices or decrease the burden of pharmacological circulatory support. Considering the high in-hospital mortality and complications in such patients, early implantation of mechanical circulatory devices capable of providing higher levels of hemodynamic support may be considered to treat these patients.
